# Does spastic myopathy determine active movement and ambulation speed in chronic spastic paresis?—A cross-sectional study on plantar flexors

**DOI:** 10.1371/journal.pone.0310969

**Published:** 2024-10-24

**Authors:** Maud Pradines, François Jabouille, Enguerran Fontenas, Idriss Baba Aissa, Caroline Gault-Colas, Marjolaine Baude, Marina Guihard, Karine Gros, Jean-Michel Gracies

**Affiliations:** 1 UR 7377 BIOTN, Laboratoire *Analyse et Restauration du Mouvement*, Université Paris Est Créteil (UPEC), Créteil, France; 2 AP-HP, Service de Rééducation Neurolocomotrice, Unité de Neurorééducation, Hôpitaux Universitaires Henri Mondor, Créteil, France; 3 Chaire "Handicap, Emploi et Santé au Travail", Université Paris-Est Créteil, Créteil, France; University of Illinois Urbana-Champaign, UNITED STATES OF AMERICA

## Abstract

**Background:**

Functional correlates of spastic myopathy, the muscle disorder of spastic paresis, are unknown.

**Objective:**

To explore reciprocal relationships between clinical and structural parameters of plantar flexors with i) ambulation speed, ii) dorsiflexion and plantarflexion torques in chronic hemiparesis.

**Methods:**

Cross-sectional trial in chronic stroke-induced hemiparesis (>6 months). Plantar flexors were quantified through i) the Five Step Assessment: maximal extensibility (X_V1_), active range of dorsiflexion (X_A_); ii) ultrasonography: fascicle length (L*f*) and thickness (Th) of medial gastrocnemius (GAS) and soleus (SOL), knee extended in an isokinetic ergometer, ankle at 80% X_V1-GAS_. Maximal isometric torques in plantar flexion (PF) and dorsiflexion (DF) and maximal barefoot 10-meter ambulation speed were collected. Relationships between structural, biomechanical, clinical and functional parameters were explored using non-parametric testing (Spearman).

**Results:**

Twenty-one subjects (age 58.0±8.4, mean±SD, time since lesion 7.8±5.7 years) were recruited, with the following characteristics: ambulation speed, 0.77±0.37m/sec; X_V1-SOL_ 92.7±10.3°; X_V1-GAS_ 91.3±9.6°; X_A-SOL_ 86.9±10.0°; X_A-GAS_ 7676±14.2°; L*f*_GAS_, 58.2±18.3mm; Th_GAS_, 17.1±3.6 mm; L*f*_SOL_, 36.0±9.6 mm; Th_SOL_, 13.8±3.3mm; PF peak-torque 46.5±34.1Nm, DF peak-torque, 20.1±19.1Nm. X_A-SOL_ and X_A-GAS_ strongly correlated with X_V1-SOL_ and X_V1-GAS_ respectively (ρ = 0.74, p = 4^E-04^; resp ρ = 0.60, p = 0.0052). Ambulation speed moderately correlated with L*f*_GAS_ (ρ = 0.51, p = 0.054), Th_GAS_ (ρ = 0.58, p = 0.02) and L*f*_SOL_ (ρ = 0.63, p = 0.009). DF and PF peak-torques both correlated with L*f*_GAS_ (ρ = 0.53, p = 0.04) a; resp. ρ = 0.71, p = 0.0015).

**Conclusion:**

In chronic hemiparesis, active dorsiflexion is mostly determined by plantar flexor extensibility. Plantar flexor fascicle shortening is associated with reduced ambulation speed and ankle torques. Attempts to restore plantar flexor extensibility might be important objectives for gait rehabilitation in chronic hemiparesis.

## Introduction

Care for patients who suffered a stroke constitutes a growing public health concern [1–3]. In chronic stages, hemiparesis causes functional impairment, reducing ambulation capacity and social participation with associated psychological hardships [4–7].

Chronic hemiparesis is due to a primarily neurological disorder, with initial agonist paresis and later emerging antagonist muscle overactivity [8, 9]. However, a second disorder, which has been individualized and named *spastic myopathy*, appears at a very early stage after the onset of paresis and paresis-induced muscle hypo-mobilization, probably long before any phenomena of motoneuronal overactivity [8–11]. Over that acute period, hypomobilization of some muscles in shortened position—in the context of paresis of the opposite muscles—generates a loss of longitudinal tension, which represents the first step of an early cascade of events involving multiple muscle transformations [12–16]. These successive changes–genetic [12], histological [13], biomechanical [14], physiological [15]—lead to clinical consequences noted as higher stiffness and extensibility loss [16]. Beyond a threshold of severity, these muscle changes increase tension within the affected muscle, thus contributing to increased muscle afferent firing and, through synaptic sensitization at the spinal level, potentially to motoneuronal overactivity and deterioration of the firing capability of the opposite motor neuron through increased reciprocal inhibition [11]. Therefore, while spastic myopathy represents a major factor limiting active movement [11], it may also play a role in further altering neurological command, a consequence that is potentially underestimated to date [8, 11, 16–22].

One frequently affected muscle group is triceps surae [11]. Even though morphometric (fascicle length, thickness) and biomechanical (stiffness) changes of gastrocnemius properties are now well identified [21–24], their putative impact on kinematic parameters, particularly during walking, have not been specifically investigated to our knowledge. Only fascicle lengths of biceps brachii and brachialis anterior, that are reduced in the chronic hemiparetic population [25–27], have been associated with upper limb function in preliminary studies [26, 27].

In addition, beyond the well-known potential relationship between maximal ankle torques and walking speed in chronic hemiparesis, the present study aimed to explore whether changes in muscle structural parameters might play a role in altering ankle torques, above and beyond the role played by alterations of the neural command to the agonist muscle [28–32]. The clarification of these relationships might help to better target the contributing factors to altered torque and poor function.

The main objectives of this study were to explore:

- the respective relations among the main clinical features of plantar flexors obtained using the Five Step Assessment, particularly with the question of the relationship between maximal active motion around a joint and extensibility of the antagonist to the intended movement [33],
- overall relationships between structural parameters (thickness, fascicle length), biomechanical capacities (maximal plantarflexion/dorsiflexion torque) and maximal ambulation speed in patients with chronic hemiparesis
- the relationship between architectural parameters and time since lesion

## Materials and methods

### Population

This cross-sectional study within the Neurorehabilitation Department at Henri Mondor University Hospitals in Créteil, France received the authorization of the Ethics Committee *CPP Sud-Est I*, *2020–061*. Patients were a convenience sample screened from clinic visits in the department and from surrounding private physical therapy offices, and recruited for this study from September 14, 2021 until December 22, 2021. All the subjects provided written consent to participate.

Inclusion criteria were: hemiparesis due to single stroke during adulthood (18 to 80 years) more than six months before enrolment, gastrocnemius hypo-extensibility (X_V1-GAS_<110°) [33] and spasticity (Spasticity Angle X_GAS_ = X_V1_-X_V3_ ≥ 5°, Tardieu) [33], ability to walk independently over 10 meters (Functional Ambulation Category score ≥4). Exclusion criteria were botulinum toxin injections in the triceps surae during the past three months before inclusion [34–36], and concurrent orthopaedic disorder around the ankle.

### Protocol

All measurements were collected during a single session and performed in the same sequential order by the same investigator, as follows.

#### 1. Clinical assessment

**Fig 1:**
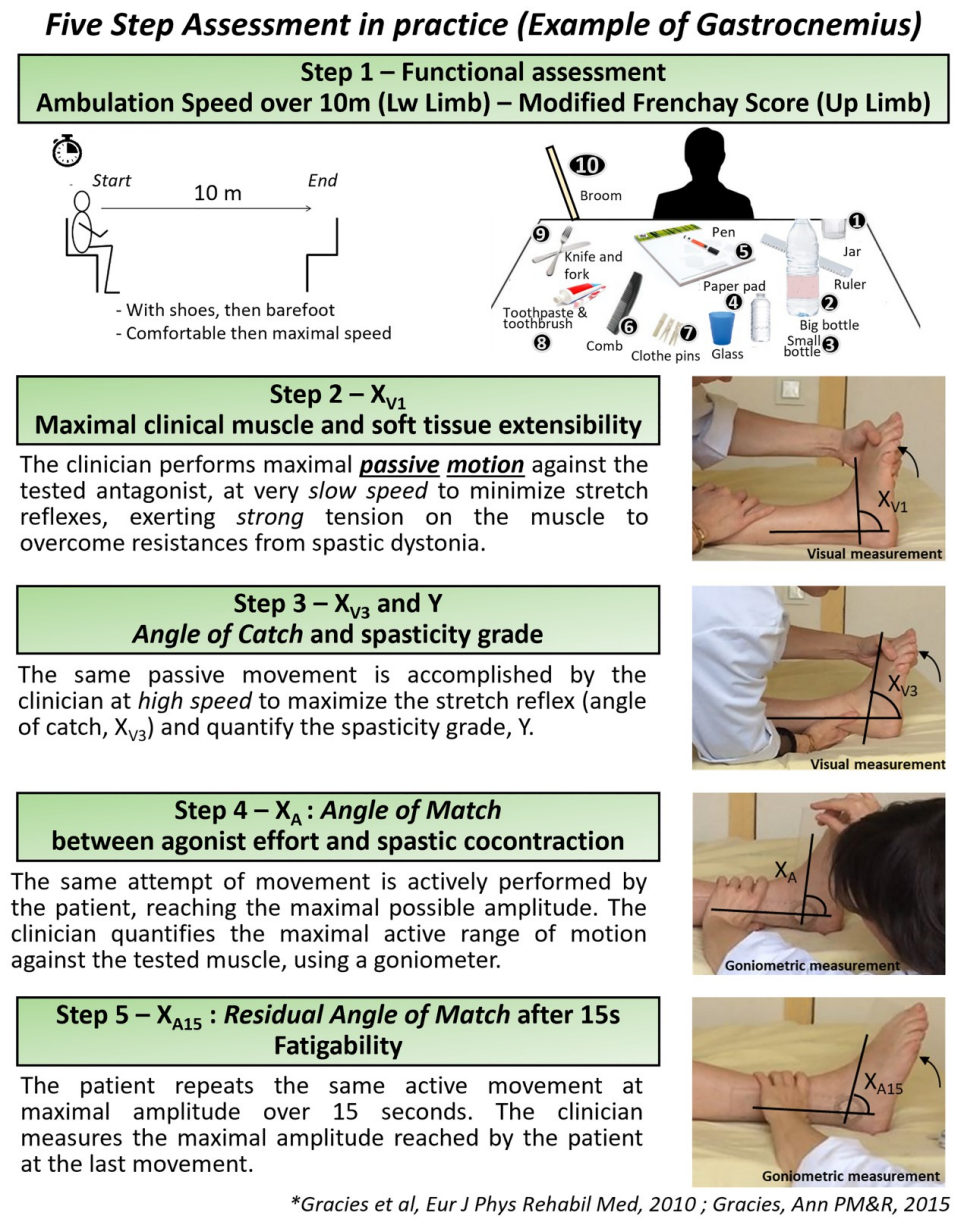
Description of the five step assessment in practice. Example of Gastrocnemius muscles.

Maximal barefoot walking speed was measured over ten meters, barefoot without technical aid, following the conditions of the AT10 (10-meter Ambulation Test) with start and end seated, which is the first step of the Five Step Assessment (FSA) [10, 33, 37]. Then, measures of maximal clinical muscle extensibility of gastro-soleus complex (X_V1-GAS_) and soleus (X_V1SOL_) were visually collected through slow and strong passive stretching movement of these muscles, subject in supine position, through a standard clinical method that has been previously described in details [38] (Fig 1). Measurements of the angles of catch (X_V3-SOL_, X_V3-GAS_) and spasticity grade (Y) were then performed. Finally, active ranges of motion against the resistances from these antagonists, i.e. angles of match between agonist efforts and antagonist passive and active resistances (X_A-SOL_, X_A-GAS_) were also quantified (Fig 1), following the steps of the FSA [33].

#### 2. Ultrasound structural assessment of the plantar flexors with EMG monitoring

Structural parameters were evaluated by an investigator blinded as the ambulation speed. Subjects were positioned in a Con-Trex^™^ isokinetic ergometer (CMV AG, Duebendorf, Switzerland) in a reproducible position with the hips flexed at 45°, the paretic knee in full extension and the paretic ankle positioned at 80% of X_V1-GAS_ (*i*.*e*. 80% of maximal clinical extensibility of the gastro-soleus complex). The paretic foot, thigh and trunk were strapped to ensure position stability during these measurements [16, 39].

In that knee-straight position, the parameters of soleus, then medial gastrocnemius, thickness and fascicle length (L*f*) were collected under passive conditions through three ultrasound images for each muscle. Prior detection of skin markings—myotendinous junction of the medial gastrocnemius (MTJ-MG), distal landmark; medial projection of the centre of the fibula head, proximal landmark—allowed standardization of the probe location (Fig 1) [24]. For collection of medial gastrocnemius muscle images, the probe was positioned midway along its body. For the soleus ultrasound acquisitions, the probe was placed directly below the MTJ-MG, so that it was seen at the edge of the screen. The probe was continuously held perpendicular to the skin, without excessive pressure to avoid deforming the tissue [40].

**Fig 2:**
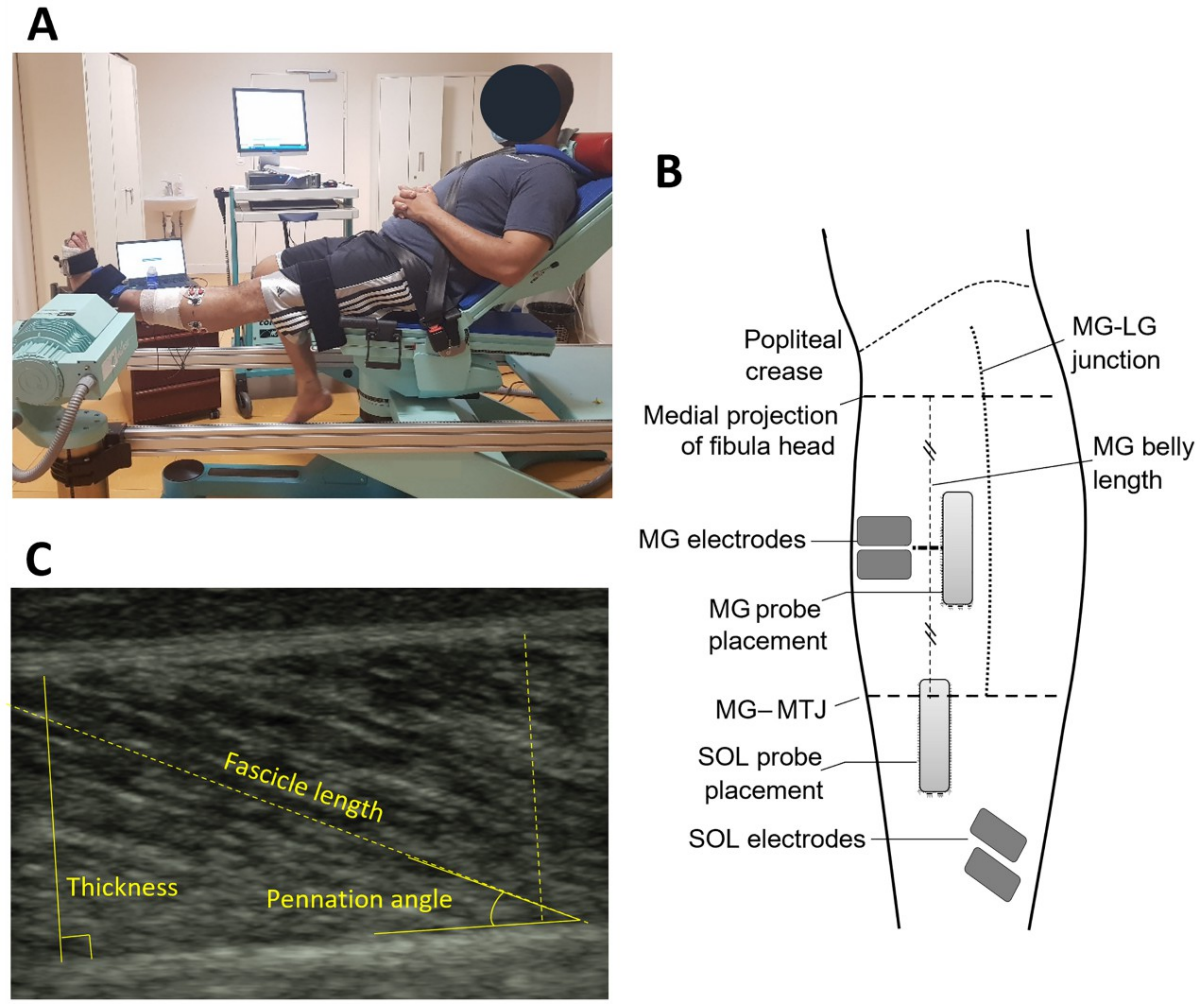
Description of the methodology process. Position during the assessment (A). Ultrasound probe placement and electrodes (B). Quantification of structural parameters through ImageJ^®^ (C).

To verify the resting state of the soleus and medial gastrocnemius during ultrasound measurements, their electromyographic (EMG) activity was collected using an EMG ME6000-T16 device (MegaElectronics Ltd, Kuopio, Finland). Subjects were first seated in a chair, the skin was abraded and two pairs of electrodes on soleus and medial gastrocnemius were positioned according to a standardized procedure following the SENIAM recommendations, while allowing for sufficient space for the ultrasound probe (Fig 2) [41]. Each subject was then positioned in the ergometer, in the position described above. Simultaneous monitoring of EMG activity of soleus and medial gastrocnemius during ultrasound acquisitions was accomplished through a trigger signal. Following the collection of ultrasound images, the subject was asked to perform a Maximal Voluntary Contraction (MVC) into plantar flexion (see below), through three consecutive trials. Soleus and medial gastrocnemius were considered at rest if EMG activity was less than 5% of the activity obtained during isometric MVC.

#### 3. Biomechanical assessment—Maximal voluntary torques around the ankle

In the position described above on the isokinetic ergometer, the subject was asked to perform maximal dorsiflexion efforts, after the above protocol that collected maximum plantarflexion torque as well. Each measurement was applied in a standardised manner including a warm-up effort before the first trial, one maximal effort to be held for five seconds, and two minutes of rest before the next trial. The best of the three peak torque measurements was used for analysis [41–43].

### Data treatment

#### Analyses of ultrasound images

The sampled acquisitions were processed by the investigator blinded as to the ambulation speed, experienced in ultrasound analyses using ImageJ^™^ software, following Mairet’s method (2006) [44]. A single acquisition was selected, based on the quality of the image (parallelism of aponeuroses, fascicles sufficiently visible). The parameters L*f*, thickness and pennation angle of medial gastrocnemius and soleus were analyzed from the best of three images (Fig 1) [21–24, 44–46].

#### EMG analysis

The electromyographic signals from soleus and medial gastrocnemius muscles were collected with a sampling rate of 1000Hz, amplified (gain x1000), saved on hard disk, and analysed using Spike2 software, version 7.02 (Cambridge Electronic Design). The signals were filtered by a FIR (Finite Impulse Response) filter with a bandwidth of 20–450 Hz, normalized by removing the drift of the DC component and then rectified with the Spike2 software. For each MVC trial, periods of 500 ms were scanned throughout the plateau and the highest mean value was chosen. The level of EMG activity selected as reference was the maximal value among the three maximal efforts. A ratio was established, considering a 500-ms period at rest during the collection of ultrasound images (250 ms on either side of the time point corresponding to the selected ultrasound image). Muscle activity during ultrasound assessments was considered at rest if it remained ≤5% of MVC [47].

### Statistical analysis

Descriptive statistics were used for continuous quantitative variables, including fascicle length, muscle thickness, dorsiflexion and plantar flexion peak torques, X_V1_, X_V3_, X_A_, ambulation speed and time since lesion. To assess correlations between the various parameters of this study, the non parametric Spearman tests were used, after removing outliers from each sample based on Z-scores >2. Statistical significance was set at 0.05 (5% alpha risk).

## Results

### Characteristics of subjects

A convenience sample of twenty-one subjects was enrolled in this study, as described in Table 1. Mean age was 58.0±8.4 years and time since lesion was 7.2±5.6 years. Maximal barefoot ambulation speed on AT10 was 0.77±0.37 m/sec. Maximal clinical extensibility of the plantar flexors was as follows: X_V1SOL_, 92.7°±10.3°; X_V1GAS_, 91.3°±9.6°. In the knee extended position described above, maximal plantar flexion torque was 46.5±34.1 Nm and maximal dorsiflexion torque was 20.1±19.1 Nm. Muscle activity during ultrasound recording at 80% X_V1_ was 6.8±6.1% of MVC for medial gastrocnemius, and 7.4±11.3% of MVC for soleus.

**Table 1:** Subject characteristics and clinical, biomechanical and structural parameters. *Ambulation speed is measured barefoot at maximal speed*. *Isometric torques were measured knee in the extended position; I*, *Ischemic stroke; H*, *Haemorrhagic stroke; BT*, *Brain Trauma*.

***Subject characteristics***	
Number	21
Age (years)	58.0±8.4
Time since lesion (years)	7.2±5.6
Gender	16M/5F
Paretic side	9L/12R
Lesion type	14I, 6H, 1BT
***Clinical parameters***	
Max ambulation speed (m/sec)	0.77±0.37
X_V1SOL_ (deg)	92.7±10.3
X_V1GAC_ (deg)	91.3±9.6
X_V3SOL_ (deg)	83.6±7.3
X_V3GAC_ (deg)	81.2±7.3
X_ASOL_ (deg)	86.9±10.0
X_AGAC_ (deg)	77.6±14.2
***Architectural parameters***	
MG fascicle length (mm)	58.2±18.3
Sol fascicle length (mm)	36.0±9.6
MG thickness (mm)	17.1±3.6
Sol thickness (mm)	13.8±3.3
***Biomechanical parameters***	
Max isometric torque in PF (Nm)	46.5±34.1
Max isometric torque in DF (N)	20.1±19.1

### Relationships between clinical parameters

X_A-SOL_ and X_A-GAS_ strongly correlated with X_V1-SOL_ and X_V1-GAS_ respectively (soleus, R = 0.74, p = 4^E-04^; medial gastrocnemius, R = 0.60; p = 0.0052, Table 2). X_V3-SOL_ and X_V3-GAS_ also strongly correlated with X_V1-SOL_ and X_V1-GAS_ respectively (soleus, R = 0.71, p = 5^E-04^; medial gastrocnemius, R = 0.61; p = 0.004, Table 2). However, X_A-GAS_ did not correlate with X_V3-GAS_ while there was some correlation between X_A-SOL_ and X_V3-SOL_ (R = 0.50; p = 0.03). Ambulation speed did not correlate with any of the individual plantar flexor parameters of the Five Step Assessment.

**Table 2:** Correlation matrix for Medial Gastrocnemius (A), and Soleus (B).

**A Med Gastroc**	1- Thickness	2- Fascicle length	3- X_V1-GAS_	4- X_V3-GAS_	5- X_A-GAS_	6- PF Peak torque	7- DF Peak torque	8- Ambulation speed
1- Thickness								
2- Fascicle length	**ρ = 0.62; p = 0.006**							
3- X_V1-GAS_	ρ = -0.19; p = 0.44	ρ = -0.05; p = 0.85						
4- X_V3-GAS_	ρ = -0.24; p = 0.36	ρ = —0.04; p = 0.88	**ρ = 0.61; p = 0.004**					
5- X_A-GAS_	ρ = 0.03; p = 0.92	ρ = 0.63; p = 0.12	**ρ = 0.60; p = 0.0052**	ρ = 0.26; p = 0.27				
6- PF Peak torque	ρ = 0.45; p = 0.18	**ρ** **= 0.71; p = 0.0015**	ρ = 0.30; p = 0.19	ρ = 0.28; p = 0.24	ρ = 0.37; p = 0.11			
7- DF Peak torque	ρ = 0.21; p = 0.38	ρ = 0.53; p = 0.04	ρ = -0.04; p = 0.88	ρ = -0.25; p = 0.31	ρ = 0.30; p = 0.20	ρ **= 0.56; p = 0.010**		
8- Ambulation speed	ρ = 0.58; p = 0.02	ρ = 0.51; p = 0.054	ρ = -0.28; p = 0.28	ρ = -0.18; p = 0.48	ρ = 0.04; p = 0.87	ρ = 0.34; p = 0.13	ρ = 0.25; p = 0.29	
**B Soleus**	1- Thickness	2- Fascicle length	3- X_V1-SOL_	4- X_V3-SOL_	5- X_A-SOL_	6- PF Peak torque	7- DF Peak torque	8- Ambulation speed
1- Thickness								
2- Fascicle length	**ρ = 0.73; p = 9** ^ **E** ^ **-04**							
3- X_V1-SOL_	ρ = -0.42; p = 0.08	ρ = -0.42; p = 0.10						
4- X_V3-SOL_	ρ = -0.02; p = 0.95	ρ = -0.07; p = 0.76	**ρ = 0.71; p = 5E-04**					
5- X_A-SOL_	ρ = -0.26; p = 0.32	ρ = -0.06; p = 0.98	**ρ = 0.74; p = 4** ^ **E** ^ **-04**	ρ = 0.50; p = 0.03				
6- PF Peak torque	*N/A*	*N/A*	*N/A*	*N/A*	*N/A*			
7- DF Peak torque	*N/A*	*N/A*	*N/A*	*N/A*	*N/A*	*N/A*		
8- Ambulation speed	ρ = 0.33; p = 0.23	**ρ = 0.63; p = 0.009**	ρ = -0.13; p = 0.61	ρ = -0.22; p = 0.37	ρ = 0.08; p = 0.76	*N/A*	*N/A*	

### Muscle architectural parameters and ambulation speed

**Fig 3:**
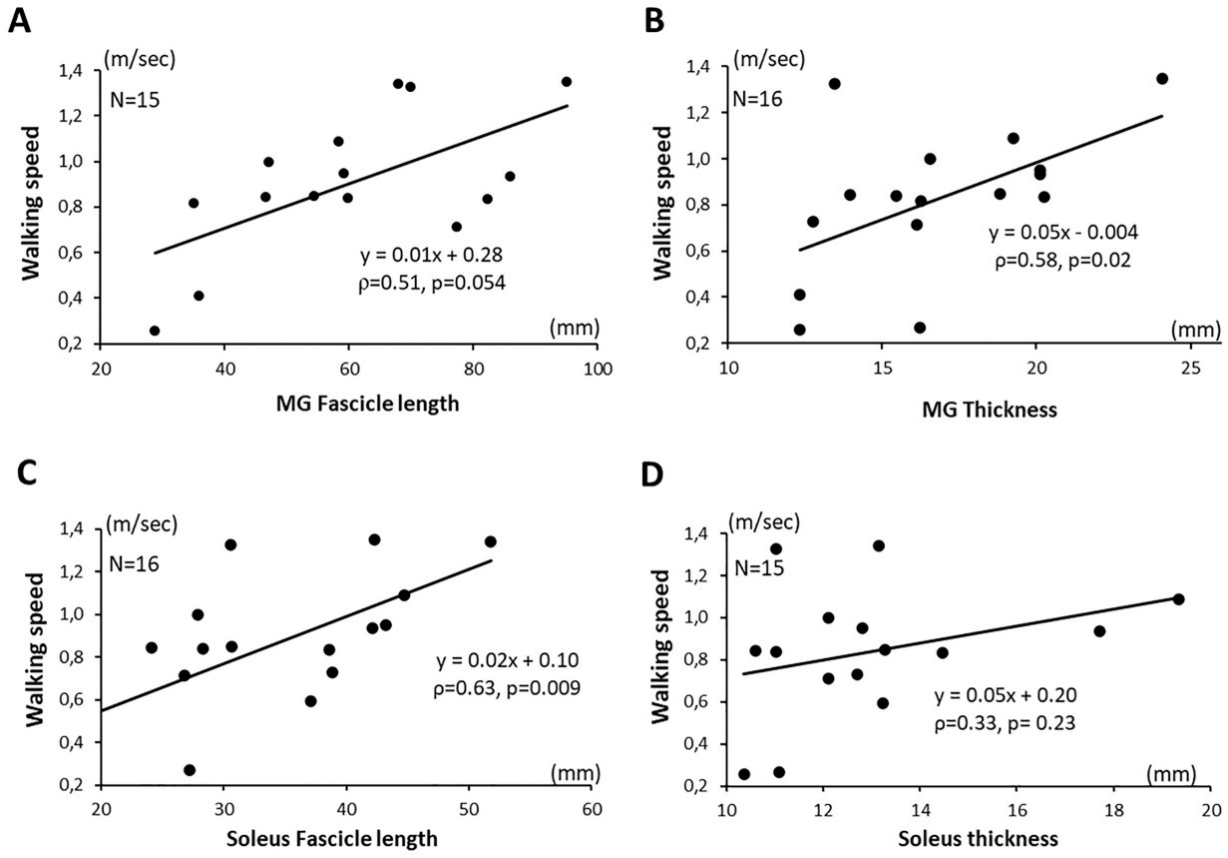
Correlations between ambulation speed and architectural parameters: i) MG Fascicle length (A), ii) MG thickness (B), iii) Soleus fascicle length (C), iv) Soleus thickness (D).

Maximum ambulation speed barefoot over 10 meters correlated with medial gastrocnemius L*f* (ρ = 0.51, p = 0.054), medial gastrocnemius thickness (ρ = 0.58, p = 0.02), and soleus L*f* (ρ = 0.63, p = 0.009). (Table 2, Fig 3). Ambulation speed did not correlate with soleus thickness (ρ = 0.33, p = 0.23).

### Relationship between MG architectural parameters and maximal torques produced around the ankle

**Fig 4:**
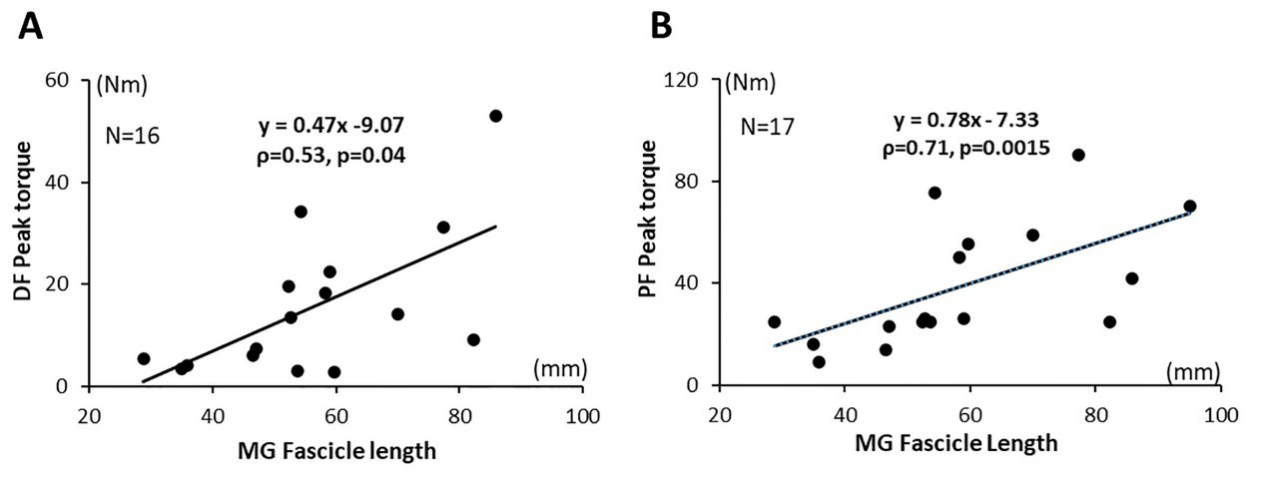
Muscle architecture and function of the muscle. Associations between DF peak-torque and MG fascicle length (A); then between PF peak-torque and MG fascicle length (B).

PF peak torque correlated with MG fascicle length (ρ = 0.71, p = 0015) and so did DF peak torque (ρ = 0.53, p = 0.04). Neither PF nor DF peak torques correlated with MG thickness (ρ = 0.45, p = 0.18; ρ = 0.21, p = 0.38, respectively) (Table 2, Fig 4).

### Other relationships between functional, biomechanical and structural parameters

**Fig 5:**
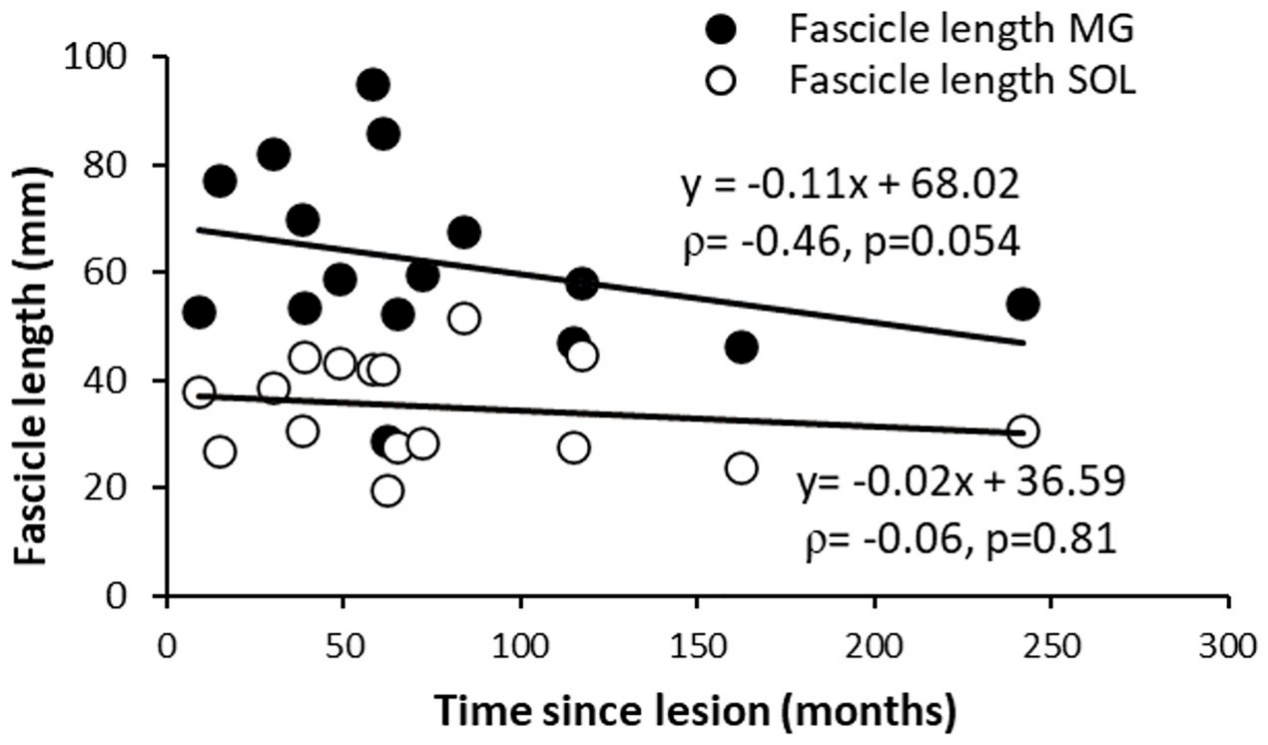
Muscle changes over time. Correlations between MG and SOL fascicle lengths, respectively, and time since lesion.

There was correlation between PF and DF peak-torques (ρ = 0.56, p<0.010; Table 2). Maximal ambulation speed over 10 meters did not correlate with PF peak torque or DF peak torque: ρ = 0.34, p = 0.13 (resp ρ = 0.25, p = 0.29; Table 2). Finally, there was a trend for a correlation between MG fascicle length and time since lesion (ρ = -0.46, p = 0.054, Fig 5).

## Discussion

This cross-sectional exploration of the correlates between the Five Step Assessment parameters and of the functional correlates of structural plantar flexor changes in chronic hemiparesis indicated that passive movement against the resistance of a given plantar flexor antagonist was a strong predictor of the active movement against that antagonist and that maximal ambulation speed moderately correlated with medial gastrocnemius and soleus fascicle lengths, respectively. This study also shows mild association between the fascicle lengths of the plantar flexors and the capacity to generate isometric force in both dorsiflexion and plantarflexion. If sufficient arguments are brought to suggest partial causality, this might provide a compelling case for the functional significance of spastic myopathy in chronic hemiparesis.

### Spastic myopathy and dysfunction: Arguments for causality

The present observations reveal strong statistical associations between passive and active clinical motions against the resistance of a given plantar flexor antagonist and mild relationships between fascicle lengths of plantar flexors and ambulation speed. We believe that major criteria favor causal relationships here [48].

First, the *strength* of these relationships is such that active movement against the passive and active resistance of a given plantar flexor antagonist is highly determined by the maximal clinical extensibility of this antagonist in the present study. This only confirms similar relationships previously shown in other samples of paretic patients, between composite passive and composite active movements in both lower and upper limbs in the adult-acquired spastic paresis population and between individual passive and active parameters in the cerebral palsy population [11, 49].

In terms of *physiological plausibility* of causal relationships, the probabilities that active movement against the resistance of a given muscle would impact passive movement and that walking speed would impact thickness and fascicle length are far lower than the opposite hypotheses, particularly when considering what is known of the relationship between architectural parameters and ankle torques, the latter factor being well-known predictors of walking speed [29, 30]. Scientific plausibility is strengthened by the coherence of the present findings with a number of other reports: it has been shown previously that walking speed was highly correlated with the composite coefficient of shortening C_SH_ in the lower limb, more so than with the coefficient of weakness (C_W_, which primarily reflects motor command) [11]. Additionally, the motor command disorder (Coefficient of weakness, C_W_) is highly related to muscle shortening (C_SH_), making it likely that the muscle disorder might worsen the impairment of the descending drive [11]. Thirdly, passive resistance from plantar flexors has long been shown to relate to active dorsiflexion angles during the swing phase in hemiparesis [50]. In patients with cerebral palsy, for which loss of muscle extensibility starts very early in life and is more severe than in adults with acquired lesions [49, 51], correlation between walking speed—resp. climb and go downstairs—and plantar flexor stiffness was also demonstrated [52, 53]. Finally, in the upper limb, fascicle length of biceps brachii and brachialis anterior, that is reduced in the chronic hemiparetic population [25–27], has also been associated with upper limb function [26, 27].

### Muscle health, an underestimated factor contributing to locomotion in spastic paresis

Data exploring relationships between muscle morphometric properties and gait are still scarce [50]. The relationship evidenced here may represent novel and potentially important data for the evaluation and treatment of patients with hemiparesis. Such data should raise the attention of clinicians to the evolving passive structural alterations of muscles previously hypo-mobilized in short position, which deserves specific treatment within the management of spastic paresis, such as the practice of high load stretching with sufficient daily durations to adequately stimulate muscle plasticity [24].

### Walking speed and maximal ankle torques, a sign of specific biomechanical strategies?

Previously, gait speed in the hemiparetic subject has been shown to correlate with dorsiflexion torques [29, 54–56]. The contrast with the present findings could involve the position in which ankle torques were measured, lack of power resulting from the small sample size, but also the fact that the present subjects were unusually fast walkers for a chronic hemiparetic population. It has been shown that walking speed involves various biomechanical strategies and muscular use in healthy [57] and paretic subjects [58–60]. While difficulty in clearing the ground in the swing phase is the major problem in slow to intermediate walkers [28], plantar flexion force becomes a dominant factor above a certain speed threshold (0.7 m/sec according to Winter) [57], conditioning propulsion [56]. Therefore, caution should be used when considering therapies such as botulinum toxin into plantar flexors for such fast walkers (>0.7 m/sec), as voluntary plantar flexor activation during the toe-off phase may be hampered by injection [61], and as a single injection could lead to residual atrophy six months to one year later [34–36, 62].

### Which disorders may hide behind the loss of torque production?

While paresis (reduced agonist activation) has been a classic factor incriminated in the loss of torque production in hemiparetic subjects [31, 63–65], the impact of intrinsic muscle properties remains scarcely explored in this population [66, 67]. Muscle thickness, and particularly fascicle length have been shown to be associated with muscle strength [68–72], therefore the influence of medial gastrocnemius fascicle length on maximal isometric plantar flexion peak torque—measured in this study with the knee in the extended position, may not be surprising. However, maximal isometric *dorsiflexion* torque also correlated with fascicle length of medial gastrocnemius here. This result may support previous findings showing negative impact of muscle shortening on the command to the opposing muscle [11, 20]. While the subject re-extends his paretic knee and tries to dorsiflex the ankle at the end of the swing phase of gait, in other words imposes increased tension on gastrocnemius muscles, the degrees of gastrocnemius overactivity and of tibialis anterior paresis gradually increase [20]. Further, as previously mentioned, it has been recently argued that spastic myopathy may play a role in the neural disorder, as severe muscle shortening may contribute to secondary descending command impairment and motoneuronal overactivity in hemiparesis, through chronically increased intramuscular tension, triggering permanent afferent firing and activity-dependent synaptic sensitization at the spinal level [11, 19, 73–76].

### Characterization of spastic hemiparesis through the Five Step Assessment?

From the present study, correlations between quantified clinical parameters and biomechanical, muscle architectural assessments support the ability of the Five Step Assessment to discriminate between the features of the syndrome of spastic paresis from clinical quantification. These results support the idea that the first technical step of this scale X_V1_ might be useful to characterize spastic myopathy and its impact on motor command, although correlation between fascicle length of both plantar flexors and their respective X_V1_ was not found in the present study, unlike previously for soleus [24]. Finally, whether against the resistance of the MG or the soleus, these data allow to reiterate that passive movements (X_V1_) might be the first contributors to active movement (X_A_), as these variables remained highly correlated in this study, confirming previous results [11, 49].

### Study limitations

The sample analyzed in this study was small for correlation analyses, thus the present findings need confirmation on a larger scale. Unwanted medial gastrocnemius and soleus activity during ultrasound recordings ‘at rest’ were above 5%, and these muscles thus could not be considered at rest: residual spastic dystonia might have been present and was in effect quantified here [19]. The comparison with healthy subjects in this context is limited [45], and characterization of spastic dystonia certainly needs further investigations [77]. Additionally, biomechanical measurement of the PF and DF peak torque, but also ultrasound quantification of soleus fascicle length and thickness were collected with the knee in the extended position. It would have been interesting to quantify these soleus parameters in a submaximal stretched position of this muscle (knee flexed), as for the medial gastrocnemius muscle. Furthermore, in these adult patients, data on height or lower limb length were not collected so walking speed could not be normalized. Finally, as previously mentioned, ambulation speed was higher in this study than the average in the general hemiparetic population [37]. Further study on this topic with slower walking patients may help explore the external validity of the present findings. Causality between muscle structural parameters on one hand and motoneuronal overactivity and function on the other hand should be demonstrated through prospective longitudinal studies from the very early days after the lesion to six months or 1 year later, to monitor muscle changes over time, and characterize the relationships of its structural components with functional, biomechanical and neurophysiological parameters.

## Conclusion

This study confirmed the critical importance of passive muscle parameters in impacting active movement capacities against their resistance in chronic spastic paresis and strongly suggested the influence of the fascicle length of the medial gastrocnemius and soleus muscles on ambulation speed in chronic hemiparetic subjects. The significance of the relationships between plantar flexor fascicle lengths, and maximal ankle torques and walking speed, supports the role of changes in intrinsic muscle properties as a likely determinant of the capacity to generate torques and functional capacities in hemiparesis. These explorations may further encourage clinicians to view the alteration of the muscle tissue in spastic paresis, spastic myopathy, as an entity for itself, requiring specific treatment through rehabilitation programs stimulating muscle plasticity.

## Abbreviations

AT10: Ten meter Ambulation Test
C_SH_: Coefficient of Shortening
C_W_: Coefficient of weakness
DF: Dorsiflexion
FAC: Functional Ambulation Category
FIR: Finite Impulse Response
FSA: Five Step Assessment
GAS: Medial Gastrocnemius muscle
L*f*: Fascicle length
MTJ: Myo-Tendinous Junction
MVC: Maximal Voluntary Contraction
PF: Plantar flexion
SOL: Soleus muscle
Th: Thickness
X: Spasticity angle
X_A_: Active range of dorsiflexion
X_V1_: Maximal clinical muscle extensibility
X_V3_: Angle of catch
Y: Spasticity grade
ρ: Spearman’s Coefficient
